# The Injury Profile of an Australian Specialist Policing Unit

**DOI:** 10.3390/ijerph13040370

**Published:** 2016-03-25

**Authors:** Brianna Larsen, Brad Aisbett, Aaron Silk

**Affiliations:** 1 Deakin University, School of Exercise and Nutrition Sciences, Geelong 3216, Australia; aaron@hpscience.com.au; 2 School of Exercise and Nutrition Sciences, Institute for Physical Activity and Nutrition (IPAN), Deakin University, Geelong 3216, Australia; brad.aisbett@deakin.edu.au; 3 Human Performance Science, Melbourne 3004, Australia

**Keywords:** police, special forces, injury analysis, workplace injury

## Abstract

This study investigated the injuries sustained by an Australian specialist police division. Injury records spanning four-years were analyzed. The role being performed when the injury occurred, injury cause, body part injured, and injury-related costs were quantified. The percentage of personnel injured multiple times was documented. One hundred and thirty eight personnel reported injuries, 58 of these on multiple occasions. This resulted in 229 injuries and 76 claims being raised. Half of the injuries occurred during operational policing tasks, however training activities accounted for >30% of injuries. The most common injury was strain/sprain, and upper body injuries were 2.5-times more common than lower-body or torso injuries. 1107 shifts were lost, and injuries cost the organization $487,159 (Australian Dollars) over the four-year period. The injury costs (both financial and in manpower) may prompt policy makers to review the current training and post-injury rehabilitation protocols.

## 1. Introduction

Police work is commonly described as one of the most dangerous and physically demanding occupations, with a higher frequency of injury occurrence relative to other professions [1,2,3,4]. Previous research investigating police workplace injury has most commonly focused on specific types of incidents, such as direct assaults on police officers [2,5,6], operations that require the use of physical force [7,8], or domestic disturbances [9,10,11]. While it is important to understand the hazards involved with such events, police officers also routinely perform tasks that do not directly involve a violent offender (e.g., driving, office duties, training tasks) [1,12]. Fewer researchers have attempted to characterize the injury profile of police officers across the breadth of tasks performed when on duty [3,12,13].

Perhaps unexpectedly given the common perception of police work, the majority of police officer injuries result from accidents such as falls, or during the performance of manual-handling tasks [1,3,12,13]. Indeed, previous studies have observed that only 9.7%–12.6% of police officer injuries across the US and Australia result from assaults or physical violence [1,12]. It is likely, then, that the narrow focus of previous literature may have grossly underestimated the frequency of police officer injury. Brandl and Stroshine [12] observed that, between 1996–1998 and 2006–2008, only 5.2% of the injuries sustained by police officers in the Milwaukee Police Department were classified as “serious”, and in most cases (52.8%) injured officers did not seek medical attention nor lose time away from work as a result of an incident (85.3%). This is in contrast to the findings by Ferguson and colleagues [1], who found that, although highly variable, the mean number of hours lost from work due to police officer injury over an eight year period (2000–2008) in Australia was 814 ± 1696 h (standard deviation; SD), with an accompanying median cost of $9900 for the organization. Sprains and strains were identified as the most common type of injury among police officers [12]. Finally, injury frequency decreased in both studies across the time periods analyzed [1,12].

While the small body of existing research affords some insight into the global injury profile of general duty police officers, there exists a higher level of police response capability [14,15] that has yet to be investigated. Examples of such specialist policing organizations include Special Weapons and Tactics (SWAT) teams in the US, and Firearms Units in the UK. These entities are specifically tasked with responding to high-risk scenarios (e.g., hostage negotiations, suicide intervention), often involving known violent offenders and in unpredictable environments [15]. Thus, members are trained in the employment of specialized weaponry and tactics, above and beyond that which can be exercised by general duties officers [16]. Given that the response capability provided by such organizations must be available 24 h per day, their operational readiness is contingent on maintaining the overall health of its members. Understanding the frequency and severity of injury in this population is therefore crucial in maintaining optimum capacity to respond to high-risk events.

This study aims to quantify the frequency and types of injury sustained by a state-based, specialist police division in Australia. Given the prevalence of injury resulting from accidents in the general policing population [1,3,12,13], injury data will be presented across all tasks performed on the job. Understanding whether many officers are being injured, or a select group of officers are being injured multiple times, may provide important organizational information regarding the suitability of the current training continuum and post-injury rehabilitation practices. Thus, the present study will also quantify the number of isolated *versus* multiple injuries in the force. To elicit an understanding of the organizational impact of workplace injuries, the current research also aims to investigate the associated costs and time away from work following a work-related injury.

## 2. Materials and Methods 

### 2.1. Data Source

The study was granted ethical approval by the Deakin University Human Research Ethics Committee (approval number: 2012-227). At the time of the study, the organization reported that 170 officers operated in the force each year (with only seven female members, or 4.1%). As the organization of interest was state-based, all reported injury data were sourced from one state within Australia. The force attended an average of 342 incidents per year from April 2010 to March 2014. As required by organizational policy, any injuries sustained whilst on shift had to be reported by officers or their superiors. If more than one officer was injured in a particular incident, separate reports were filed for each individual. There were 42 events across the observation timeframe that were recorded but did not progress to an injury/incident classification. While the reason for this non-progression is not fully understood, it is possible that they were classified as “near misses” (and thus, resulted in no actual physical or psychological injury). These events have not been included in the final analysis. No other injury data collected during the four-year timeframe were excluded from the study.

### 2.2. Data Collection

The data were collected by the organization via the use of a specialized injury report software system. An administrative employee for the organization extracted the relevant data as an Excel spreadsheet, which was provided to the research team. De-identified injury report data spanning four years (April 2010–March 2014 inclusive) were analyzed. Injury data were collected prior to this date, however the organization underwent significant structural changes that altered the operational duties performed by its members. Thus, injury claims submitted prior to April 2010 may not reflect the current training and operational tasks performed by personnel. Additionally, the current injury recording system was introduced in April 2010; therefore, all injuries in the observation timeframe reflect a consistent data source.

### 2.3. Variables

For the purpose of the study, injury was defined as any mild physical harm (e.g., bruises), or any major physical harm involving outpatient or inpatient treatment [17]. The specific variables of interest in the current study were: the frequency and types of injury sustained, the role/duty being performed when the injury occurred, the cause of the injury (or “injury attribution”), the main body part injured, and whether the incident resulted in a claim being submitted. In the case of the claimed injuries, additional information was provided to allow for the calculation of injury-related costs (medical, hospital, legal, and wage costs), as well as the time spent away from work (e.g., number of shifts lost). The sex of the injured employee was also provided. Furthermore, the (de-identified) codes corresponding to each injury report allowed the research team to identify the personnel who were injured multiple times across the observation time frame, and separate these from the isolated injuries (*i.e.*, personnel who sustained just one injury across the four years). The number of injuries per person in the “injured multiple times” group was calculated by dividing the total number of injuries sustained by the number of personnel who sustained multiple injuries.

The data categories provided by the organization were used by the research team as a starting point when creating the categories utilized in the final analysis. As an example, the original dataset contained 39 possible injury sites under the “main body part injured” headline. Including all 39 sites for analysis was considered excessive by the research team, as at times the difference between certain body parts was small (e.g., “neck” and “neck muscles”). Thus, for brevity these sites were distilled into 11 slightly broader categories. The first author was responsible for distilling the original dataset into relevant categories, and this was then crosschecked and approved by the other researchers. The same process was undertaken for all other categories (*i.e.*, type of injury, injury attribution *etc.*) to ensure that the analysis quantified the injury profile of officers in a way that was appropriate and meaningful.

The type of injury was classified as: fracture/dislocation, open wound, exposure (e.g., blood, saliva), strain/sprain, workplace stress, superficial injury (e.g., cuts, bruises), or other/not assigned. Roles being performed at the time of injury included: operational policing job, training activity, office work, or other/not assigned. Injury attribution categories were: physical interactions with a non-compliant offender, routine duties (e.g., office work, manual handling), training (operational scenarios), training (general fitness), slip/trip/fall, workplace stress (e.g., mental health issues), work equipment, vehicle accident, or other/not assigned. Finally, the “main body part injured” was categorized as: head/neck, shoulder, arm, hand/wrist, chest/ribs/torso, back, hip, leg, foot/ankle, psychological, or other/not assigned.

The financial costs associated with each claimed injury were supplied by the organization. The original dataset received by researchers was broken down into medical costs, hospital costs, legal costs, wage costs, and other costs. Individual line items in the excel spreadsheet were tallied in order to generate a total under each of the category headings, which are presented in the Results. The range of responses (e.g., the lowest to the highest cost) has also been presented in order to highlight the variation in the incurred costs between different injuries. All costs are presented in Australian Dollars ($AUD). In addition, the number of shifts lost as a result of injury were recorded by the organization and analyzed to give a more comprehensive idea of the operational burden of injury. Given the purposes of this study, simple descriptive statistics were used in the data analysis. All data is presented as totals, ranges, and percentages.

## 3. Results

A total of 229 injury incidents were analyzed across the four-year observation timeframe, which equates to 134.7 injuries per 100 employees. The vast majority of injuries were suffered by males (94.9%), which is not unexpected due to the male-dominated demographic of this vocation (95.9% of the current population). Information regarding personnel injury frequency can be found in Table 1.

The descriptive analysis of injury type, injury attribution, duty at the time of injury, and the main body part injured is presented in Table 2. Whilst almost half of all reported injuries over the four-year period occurred when personnel were on an operational policing job, training activities (either operational or fitness-based) accounted for more than 30% of the total injuries (Table 2). By far the most predominant injury type reported was strain/sprain. Injuries to the upper body were experienced 2.5-times more frequently than injuries to either the lower body or torso (including back). Finally, injuries associated with workplace stress arose from a mix of stimuli, including acute or chronic exposure to critical incidents (e.g., a shooting, homicide), office stress, and bullying.

### 3.1. Strains and Sprains

**Figure 1:**
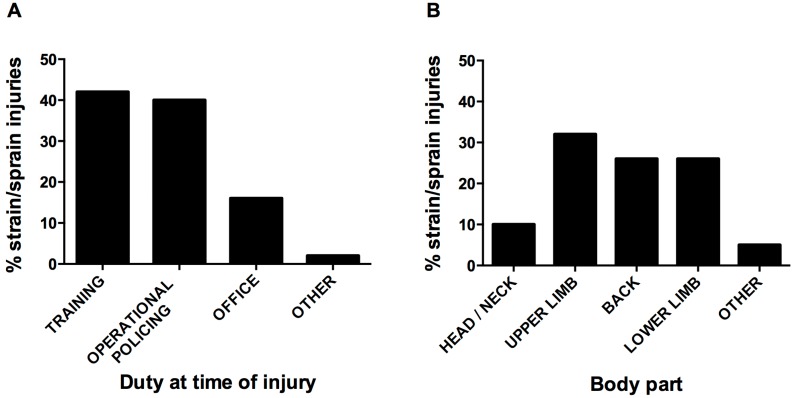
Strain/sprain injuries by duty type at the time of injury (**A**) and body part (**B**).

Due to the observed dominance of the strain/sprain injury type, a dedicated analysis was conducted. Figure 1 provides a breakdown of strain/sprain injuries by duty type (A) and body part (B). For the purpose of the figure, training (operational) and training (fitness) have been combined into a single “training” category. “Operational policing” has been used to describe any duties outside the office, whereas “office” reflects typical office duties (*i.e.*, administration tasks). For brevity, the body part injured categories have also been condensed (from the categories provided in Table 2).

### 3.2. Cost of Injury

The analysis of claimed injuries, and the associated time away from work and financial costs, is presented in Table 3. A third of injuries suffered by personnel resulted in a claim being submitted. During the four-year period 1107 work shifts were lost due to injury, with an average of 26 shifts lost per claim (*n* = 49; range 1–146). Of the 76 claims submitted, 58 (76%) were for strain/sprain injuries. These injuries accounted for 92% of the total shifts lost and associated injury costs. Office strain/sprain injuries (predominantly manual handling in nature) resulted in 286 lost shifts and cost the organization in excess of $125,000. However, it is also important to note that 17 of the claimed injuries did not result in any organizational costs.

## 4. Discussion

During the four years investigated, 138 personnel reported being injured, resulting in 229 injuries and 76 claims being submitted. Of the 138 injured personnel, 58 reported being injured multiple times. The most dominant injury type was strain/sprain, accounting for over 60% of reported injuries. In total, 1107 shifts were lost to injury and the financial burden to the organization was $487,159.

Relative to past Australian research, the incidence of injuries arising from non-compliant offenders was high. Previously, it has been shown that only 12.6% of general duties police officer injuries result from occupational violence [1], whereas the present research observed that 31.4% of injuries resulted from dealings with non-compliant offenders, making it the leading cause of injury attribution in this cohort. However, this finding is not unexpected; specialist police forces in Australian are often deployed in place of general duties officers to contain unpredictable and/or violent offenders [16]. It is reasonable to assume that as exposure to hostile offenders increases, so too would injuries resulting from offender contact or assault. While the increased incidence of violence-related injuries relative to past research is perhaps not surprising given the population of interest, no previous research had quantified the injuries sustained by a specialist police force. Data from the US has found that, while only 9.7% of police officer injuries arose directly from assault, 41.7% injuries were attributed to a “resisting subject” [12]. Inconsistencies across international injury profiles may result from a number of factors, including differences in the rate of criminal activity in the area, or variations in the duties performed by different subsets of the policing population. Therefore, whilst evaluating international injury data may be important in contextualizing the extent of a problem, obtaining local data is paramount when the information will be used to modify existing workplace practices. The fact that 68.6% of injuries in the current data set occurred whilst performing non-offender related tasks does, however, validate the inclusion of all work tasks when conducting a comprehensive occupational injury analysis.

In concert with past research [12,18], strain/sprain was found to be the most predominant injury type among officers. However, the percentage of strain/sprain injuries (relative to other types of injury) in the present study (61.1%) was considerably higher than past research. Brandl and Stroshine [12] and Reichard and Jackson [18] reported that, although strain/sprain was the most common type of injury among police officers, it accounted for 19.5% and 34% of all injuries, respectively. The current study observed that it was equally likely for specialist police personnel to experience a strain/sprain injury during an operational policing job as during training (whether that be operationally-focused or geared toward physical fitness development). While it could be argued that some injuries are inevitable when working in an unpredictable and hazardous occupation, the substantial burden of training injuries in the current cohort suggests that improvements could be made to improve the safety of officers during training exercises.

Given the size of the population in question, the 1107 shifts lost and $487,159 in financial costs incurred over the four-year period placed considerable strain on the organization. Ferguson *et al.* [1] found that between 2000 and 2008, injured Australian general duties officers incurred a median cost of $9900 and missed 587–814 h of work (~73–102 shifts) on average, depending on the type of injury. By comparison, the current analysis found that the time away from work was considerably smaller (an average of 26 shifts lost), however the costs associated with the injury were higher, totaling $13,946 on average. In addition to the financial costs, the logistics involved with replacing injured officers, organizing appropriate treatment, and conducting “back-to-work” testing are time-intensive. Further, in a relatively small, specialist cohort, each injured operator can have a substantial adverse impact on their working crew. Each of these reasons should provide added incentive for organizations to reduce the frequency and severity of injury amongst their personnel.

An area of particular concern highlighted by the present results is the number of officers that reported multiple injuries during the four-year timeframe. Fifty-eight officers were injured 2.6 times (on average), making up 65.1% of the total injuries. This finding invites speculation as to whether the recovery time and/or rehabilitation processes utilized by officers was adequate in allowing full recovery from sustained injuries. While analyzing such factors is outside the scope of the current dataset, it is possible that officers are returning to duty when they are not fully rehabilitated—a factor that may increase their risk of sustaining additional injuries. Indeed, there is evidence in other occupational populations that prior injuries represent an independent risk factor for sustaining subsequent injuries in the workplace [19,20,21,22]. If this is the case in the current cohort, future research should endeavor to determine the suitability of the current rehabilitation and “return to work” protocols. It was also observed that personnel in the present study were just as likely to be injured whilst on an operational policing job as during a training activity (operational or fitness). While the non-compliant offender injuries could be considered a hazard of the occupation, the high rate of training injuries may reflect a problem with the current training regime. The challenge for such organizations is to provide personnel with training that reflects the sometimes-hazardous aspects of the job, while balancing this “operational preparedness” against injury mitigation.

It should be noted that officers in the present study were in some instances responsible for completing their own injury reports (e.g., for minor injuries), which may slightly increase the chance of reporting bias (e.g., officers may downplay the severity of an injury to avoid being taken off shift, or exaggerate the seriousness of their injury to increase compensation or time away from work). An additional limitation of the dataset is that only the “main body part injured” was recorded by the organization, which may mean that secondary injuries sustained during the same incident were not captured. If anything, this means that the number of injuries recorded may underestimate the total number of injuries sustained by this cohort. It is, however, unlikely that any serious injuries would have gone unreported by the organization. Despite these limitations, the current findings reflect a significant step forward in quantifying the injuries sustained by specialist police forces when on duty.

## 5. Conclusions 

Between April 2010 and March 2014, 138 state-based specialist police personnel reported a workplace injury, 58 of these on multiple occasions. This resulted in 229 reported injuries, with 76 claims being raised. Strain/sprain was by far the most dominant injury type, accounting for more than 60% of injuries. In total, 1107 shifts were lost to injury, and the financial costs to the organization totaled $487,159. While dealing with violent offenders is inherently hazardous, training and office activities accounted for half of all injuries sustained. This finding suggests that more focus should be placed on minimizing ”avoidable” injuries within the force. Modifying the injury rehabilitation programs may also help minimize the number of personnel sustaining multiple workplace injuries, although further research into the suitability of the current rehabilitation protocols is required.

## Figures and Tables

**Table 1:** Descriptive analysis of personnel injured during the April 2010–March 2014 period.

Personnel Injured	*n*	%
Male	131	94.9
Female	7	5.1
**Total**	138	100
**Isolated injuries**		
Injuries	80	34.9 (of total injuries)
Personnel impacted: Male (*n* = 74), Female (*n* = 6)	80	58.0 (of injured personnel)
**Injured multiple times**		
Injuries	149	65.1 (of total injuries)
Personnel impacted: Male (*n* = 57), Female (*n* = 1)	58	42.0 (of injured personnel)
Average injuries per employee	2.57	-

**Table 2:** Descriptive analysis of injury prevalence, duty at the time of injury, injury type and attribution, and body part afflicted.

Year (1 April to 31 March)	Injury Count	% of total
2010–2011	35	15.3
2011–2012	59	25.8
2012–2013	69	30.1
2013–2014	66	28.8
**Total**	**229**	**100**
**Duty at time of injury**		
Operational policing	112	48.9
Training activity	76	33.2
Office work	38	16.6
Other/not assigned	3	1.3
**Injury attribution**		
Non-compliant offender	72	31.4
Routine duties	48	21.0
Training (operational)	40	17.5
Training (fitness)	30	13.1
Slip/trip/fall	12	5.2
Workplace stress	11	4.8
Work equipment	9	3.9
Other/not assigned	4	1.7
Vehicle accident	3	1.3
**Injury type**		
Strain/sprain	140	61.1
Superficial injury	32	14.0
Exposure	28	12.2
Workplace stress	11	4.8
Open wound	10	4.4
Fracture/dislocation	8	3.5
**Body part**		
Hand/wrist	48	21.0
Back	38	16.6
Head/neck	37	16.2
Leg	25	10.9
Shoulder	19	8.3
Other/not assigned	18	7.9
Foot/ankle	15	6.6
Psychological system	11	4.8
Arm	10	4.4
Chest/ribs/torso	5	2.2
Hip	3	1.3

**Table 3:** Injury claims submitted and associated financial costs during the four-year period.

Claim Submitted	*n*	% of Total
No	153	67
Yes	76	33
**Costs**	**$AUD**	**Range**
Average medical cost (*n* = 42)	$2815	$103–$7960
Total medical costs	$107,276	
Average hospital cost (*n* = 11)	$1334	$516–$2486
Total hospital costs	$13,259	
Average legal cost (*n* = 16)	$641	$47–$2572
Total legal costs	$10,184	
Average wage cost (*n* = 48)	$8604	$271–$42,609
Total wage costs	$353,130	
Average “other” costs (*n* = 6)	$552	$16–$1079
Total “other” costs	$3310	
**Total accumulated costs**	**$487,159**	****
